# Association between maternal smoking history and congenital anomalies in children: Results from the Japan Environment and Children's Study

**DOI:** 10.1111/cga.12430

**Published:** 2021-06-08

**Authors:** Akiko Tsuchida, Kei Hamazaki, Mika Kigawa, Tomomi Tanaka, Mika Ito, Hidekuni Inadera, and the Japan Environment and Children's Study Group

**Affiliations:** ^1^ Faculty of Medicine, Department of Public Health University of Toyama Toyama Japan; ^2^ Toyama Regional Center for JECS University of Toyama Toyama Japan; ^3^ Department of Public Health Graduate School of Medicine, Gunma University Maebashi Gunma Japan; ^4^ Department of Liberal Arts and Human Development Kanagawa University of Human Service Yokosuka Kanagawa Japan; ^5^ Faculty of Medicine, Department of Pediatrics University of Toyama Toyama Japan; ^6^ Faculty of Medicine, Department of Obstetrics and Gynecology University of Toyama Toyama Japan

**Keywords:** birth cohort, birth defects, congenital malformation, tobacco smoke, trisomy

## Abstract

We investigated the relationship between maternal smoking history and congenital anomalies in children. Drawing on data from the Japan Environment and Children's Study collected between January 2011 and March 2014, the smoking habits of pregnant women were categorized as “never smoked,” “quit before pregnancy, “quit after pregnancy,” and “full smoking.” Of the 91 626 participants examined, a total of 2199 (2.4%) infants were born with any congenital anomalies. Logistic regression analysis was used to determine the odds ratio for congenital anomalies in each group based on maternal smoking history. No significant difference was seen between the full‐smoking and never smoked groups in the odds ratios for congenital anomalies of the nervous system; the eyes, ears, face, and neck; the cardiovascular system; or the musculoskeletal system. However, in the full‐smoking group, the odds ratios for trisomy (adjusted odds ratio, 2.14; 95% confidence interval, 1.15‐3.97) and any congenital anomalies (adjusted odds ratio, 1.35; 95% confidence interval, 1.09‐1.67) were significantly higher compared with the never smoked group. Our results indicate that continuing to smoke during pregnancy is associated with increased risk of trisomy and any congenital anomalies in the general Japanese population.

## 1 INTRODUCTION

According to the World Health Organization, approximately 50% of all congenital anomalies (CAs) cannot be associated with a specific cause, but smoking tobacco during pregnancy is a possible risk factor for CAs and should be avoided by pregnant women.^1^

Of the main components of tobacco smoke, nicotine has been identified as a nervous system teratogen^2^ and carbon monoxide has been indicated as a possible teratogen.^3^ Tobacco smoke contains over 7000 chemical compounds, hundreds of which have harmful effects in humans.^4^ Therefore, it is important to investigate possible associations between CAs and smoking and/or exposure to tobacco smoke during pregnancy.

According to a meta‐analysis^5^ of observational studies conducted between 1959 and 2010, high‐incidence CAs correlated with maternal smoking during pregnancy include congenital heart defects, musculoskeletal system abnormalities, amyelia, hyperdactyly, hypodactyly, clubfoot, craniosynostosis, facial abnormalities, ocular abnormalities, cleft face, lip, and palate, gastrointestinal abnormalities, gastroschisis, anal atresia, hernias, and cryptorchidism. Other meta‐analyses investigating the association between maternal smoking during pregnancy and CAs found a statistically significant association between maternal smoking and elevated risks for specific CAs, including cleft lip,^6^, ^7^ congenital heart defects,^8^, ^9^ cryptorchidism,^10^ and neural tube defects.^11^ Still other meta‐analyses investigating the association between CAs and maternal exposure to second‐hand smoke during pregnancy found elevated risks of cleft lip^12^ and neural tube defects.^13^ However, other meta‐analyses examining a possible association between maternal smoking during pregnancy and Down syndrome found no apparent association.^14^

In a Danish register‐based birth cohort study of 838 265 singleton liveborn babies, there was a significantly higher rate of CAs, including oral clefts and respiratory and cardiovascular abnormalities, in children born to women who had smoked during pregnancy.^15^ Moreover, among 1 413 811 infants registered with the Swedish Health Registries, there were significantly higher rates of “any malformations” among children born to women who had smoked during pregnancy.^16^ However, no large‐scale birth cohort study investigating the association between CAs and maternal smoking during pregnancy has been conducted in Japan or elsewhere in Asia. In 2012, the average smoking rate among Japanese women in their 20s and 30s was 12.3% and 11.9%, respectively.^17^ These are relatively high rates of smoking among women of reproductive age in Japan.

In this study, we examined data of approximately 100 000 pregnant women who participated in the Japan Environment and Children's Study (JECS), a nationwide birth cohort study, to investigate the associations between CAs in infants and maternal smoking behavior in the early stages of pregnancy.

## 2 MATERIALS AND METHODS

### 2.1 Study design

The JECS is a birth cohort study (conducted principally by the Japanese Ministry of the Environment) investigating associations between environmental factors and childhood health and development. Recruitment for the study was carried out at 15 regional centers in Japan from 2011 to 2014. Participant recruitment involved a face‐to‐face explanation of the survey to pregnant women, after which self‐administered informed consent was obtained. Further details of the JECS study design have been reported elsewhere.^18^, ^19^

**FIGURE 1:**
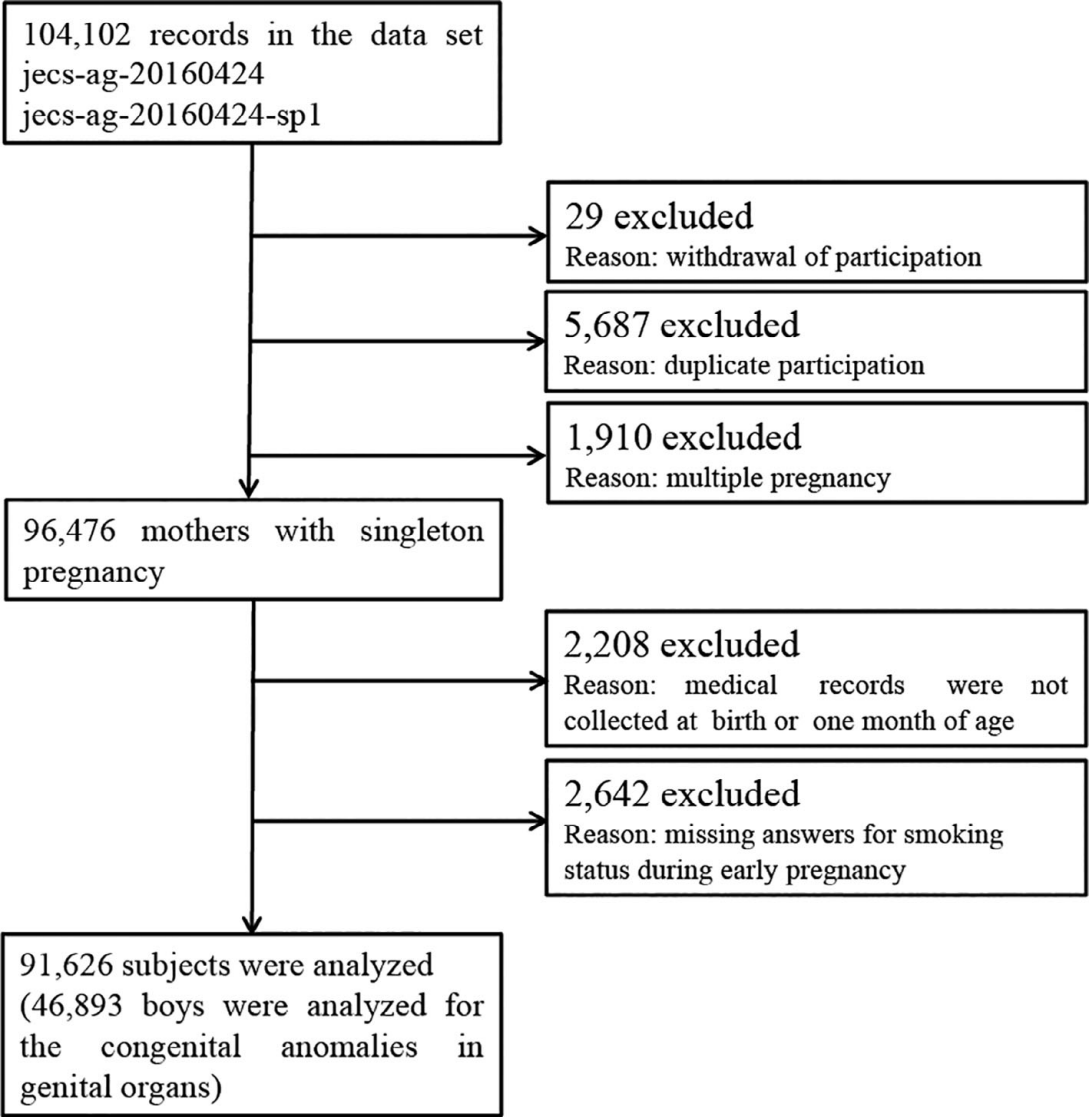
Flow diagram of the enrollment and exclusion process in this study

The present study analyzed data from the “jecs‐ag‐2016042” and “allbirth_revice001_ver001” datasets, both of which include data of 104 102 fetuses and their mothers. The final analysis included data on 91 626 participants after excluding those with missing data about smoking during the early stages of pregnancy, those who withdrew consent, those with multiple consents for multiplicate participation (after the second instance), those with multiple births, and those with missing data in transcripts of medical records at birth and at the 1‐month follow‐up (Figure 1).

The JECS protocol was reviewed and approved by the Ministry of the Environment's Institutional Review Board on Epidemiological Studies and the Ethics Committees of all participating institutions.

#### 2.1.1 Questionnaires about Exposure to Tobacco Smoke

Self‐administered questionnaires about smoking habits were distributed to and collected from participants by the study's research staff during early pregnancy. Smoking habits were categorized into four possible responses as follows: “never smoked,” “quit before pregnancy” (QBP), “quit after pregnancy” (QAP), and “full smoking.” Participants with a smoking history were asked about the age they started smoking and the approximate number of cigarettes they smoked per day on average, and those who had quit were asked when they quit. Based on these answers, the number of years of smoking and the pack‐years were calculated.

The questionnaire included the following item on the frequency of tobacco smoke exposure before pregnancy: “Before the present pregnancy, how many times per week did you encounter tobacco smoke from others, either within buildings outside the home, in the home, or at the workplace?” Other items covered the smoking behavior of the child's father, the number of smokers in the family, and the number of smokers around the mothers during the daytime.

#### 2.1.2 Main outcomes

Using hospital chart histories recorded during childbirth and at 1‐month follow‐up examinations, categorical data on 61 types of CAs were recorded in the questionnaires.^20^ We selected the following 31 ailments that were easily identified at birth and necessitated clinical responses: anencephaly, encephalocele, hydrocephaly, holoprosencephaly, spina bifida, ablepharon, anophthalmos, congenital cataract, facial cleft, cleft palate, cleft lip, cleft lip and palate, esophageal atresia, intestinal atresia, duodenal atresia, anorectal atresia, cryptorchidism, hypospadias, polydactyly of fingers or toes, syndactyly of fingers or toes, cleft hand, cleft foot, diaphragmatic hernia, omphalocele, gastroschisis, trisomy 13, trisomy 18, and Down syndrome.

Data on outcomes were collected on two occasions: at birth and during the 1‐month follow‐up examination. In the event of a contradiction between the two time points, a CA was accepted as being present if the CA was indicated at either time point, except in the case of omphalocele. Omphalocele at the 1‐month follow‐up occurred 10 times more than expected. This might have resulted from the fact that the Japanese word for “omphalocele” is often confused with that for “umbilical hernia.” Therefore, we included omphalocele only when it was detected at birth. We determined the odds ratio (OR) to investigate the association of CA with smoking by using the main CA groups from the ICD‐10 classification system. Of the genital organ CAs, cryptorchidism and hypospadias can occur in only male children, so the analysis of these conditions was performed for the 46 893 male children included in the study.

In addition, among the anomalies for which information was collected by the Japan Association of Obstetricians and Gynecologists (JAOG) survey, anomalies observed in more than 50 cases in this study were analyzed separately without grouping (Table 1). Although each disease included in the congenital heart disease category is a common congenital abnormality (eg, atrial septal defect, ventricular septal defect), we could not analyze individual diseases because the items on the transcription sheet in this study were for “congenital heart disease,” and were not listed by their individual disease names. The individual diseases analyzed in this study are as follows: hydrocephaly, cleft palate, cleft lip, cleft lip and palate, cryptorchidism, hypospadias, polydactyly of fingers, polydactyly of toes, syndactyly of toes, and Down syndrome.

**TABLE 1:** Congenital anomalies according to maternal smoking history

	ICD10 Code	Total	per 10 000 pregnancies	Never smoked	Quit before pregnancy	Quit after pregnancy	Full smoking
Nervous system (Q00‐07)		173	(18.9)	100	43	18	12
Q00	Anencephaly	24	(2.6)	17	4	2	1
Q01	Encephalocele	19	(2.1)	11	4	2	2
Q03	Hydrocephaly	81	(8.8)	44	19	10	8
Q04.2	Holoprosencephaly	33	(3.6)	21	8	3	1
Q05	Spina bifida	32	(3.5)	15	11	4	2
Eyes, ears, face, and neck (Q10‐18)		63	(6.9)	38	13	8	4
Q10	Ablepharon	13	(1.4)	7	3	3	0
Q11	Anophthalmos	22	(2.4)	12	6	3	1
Q12.0	Congenital cataract	25	(2.7)	16	4	3	2
Q18.8	Facial cleft	8	(0.9)	6	0	1	1
Cardiovascular system (Q20‐28)		1058	(115.5)	616	239	139	64
Oral clefts (Q35‐37)		229	(25.0)	128	58	33	10
Q35	Cleft palate	51	(5.6)	34	11	5	1
Q36	Cleft lip	74	(8.1)	38	20	10	6
Q37	Cleft lip and palate	116	(12.7)	63	28	21	4
Digestive system (Q38‐45)		91	(9.9)	55	31	2	3
Q39.0	Esophageal atresia	21	(2.3)	9	10	1	1
Q41	Intestinal atresia	16	(1.7)	9	7	0	0
Q41.0	Duodenal atresia	17	(1.9)	11	5	0	1
Q42.0	Anorectal atresia	44	(4.8)	31	11	1	1
Genital organs (Q50‐56)		328	(69.9)	190	79	38	21
Q53	Cryptorchidism	279	(59.5)	161	68	32	18
Q54	Hypospadias	58	(12.4)	33	15	7	3
Musculoskeletal system (Q65‐79)		332	(36.2)	185	86	38	23
Q69	Polydactyly of fingers	105	(11.5)	57	27	12	9
Q69	Polydactyly of toes	83	(9.1)	46	21	12	4
Q70	Syndactyly of fingers	43	(4.7)	20	16	3	4
Q70	Syndactyly of toes	90	(9.8)	53	22	9	6
Q71.6	Cleft hand	5	(0.5)	4	1	0	0
Q72.7	Cleft foot	7	(0.8)	6	1	0	0
Q79.0	Diaphragmatic hernia	40	(4.4)	25	10	3	2
Q79.2	Omphalocele^a^	35	(3.8)	19	9	4	3
Q79.3	Gastroschisis	14	(1.5)	6	5	2	1
Trisomy (Q90‐91)		182	(19.9)	110	40	14	18
Q90	Down syndrome	139	(15.2)	85	30	11	13
Q91.0	Trisomy 18	38	(4.1)	21	8	4	5
Q91.4	Trisomy 13	7	(0.8)	5	2	0	0
Any congenital anomalies		2199	(240.0)	1263	526	273	137

^a^ All figures are counted from medical records at birth only.

#### 2.1.3 Statistical Analysis

Maternal age at delivery was categorized as <25, 25‐29, 30‐34, 35‐39, and ≥40 years. Body mass index (BMI) before pregnancy was categorized as <18.5 kg/m^2^, ≥18.5 to <25 kg/m^2^, and ≥25 kg/m^2^. Marital status was classified as “married” or, in the case of unmarried, divorced, or widowed mothers, “single.” Education history was categorized as <13 years, 13‐14 years, and ≥15 years. Yearly income was categorized as <¥4 000 000, ≥¥4 000 000 to <¥6 000 000, and ≥ ¥6 000 000. Alcohol intake was categorized as “never drank,” “quit before pregnancy,” and “full drinking.” Based on the charts, we recorded “yes” for spontaneous pregnancy,” “no” for “induction of ovulation,” and “artificial insemination by husband” for “in vitro fertilization,” “micro‐fertilization,” “embryo transplantation,” “frozen embryo transplantation,” and “blastocyst implantation.” Folic acid intake was categorized as “yes” if the participant answered “once a month or more” on the early pregnancy questionnaire, and the response “don't take it” was categorized as “no.”

To identify the association between CAs and maternal smoking, we performed a logistic regression analysis and determined the 95% confidence intervals (CI). In the multivariable logistic regression analysis, we adjusted for maternal age at delivery, pre‐partum body mass index, history of diabetes mellitus, marital status, education history, annual household income, in vitro fertilization or artificial insemination, alcohol intake, frequency of tobacco smoke exposure, folic acid intake, antihypertensive intake, anti‐convulsant intake, and retinoic acid intake during early pregnancy. All statistical analyses were performed using SAS version 9.4 software (SAS Institute Inc., Cary, NC).

## 3 RESULTS

Of the 91 626 participants included in our analysis, 2199 (2.4%) had CAs (any CAs).

Table 1 shows the number and frequency of CAs per 10 000 births according to maternal smoking behaviors during pregnancy. The most common CAs were cardiovascular anomalies, with 1058 cases (115.5 CAs per 10 000 births) recorded. Other CAs included 328 genital organ CAs (69.9 per 10 000 male births), 332 musculoskeletal CAs (36.2 per 10 000 births), 229 oral clefts (25.0 per 10 000 births), 182 trisomy (19.9 per 10 000 births), 173 CAs related to the nervous system (18.9 per 10 000 births), and < 100 CAs related to the digestive system and the eyes, ears, face, and neck (9.9 and 6.9 per 10 000 births, respectively).

Categorized by smoking habit, “full smoking” was associated with indices of lower socioeconomic status (eg, BMI, marital status, education history, and annual household income) compared with the other groups (Table 2).

**TABLE 2:** Characteristics according to congenital anomalies (*N* = 91 626)

	Never smoked		Quit before pregnany		Quit after pregnancy		Full smoking	
	53 356	58.2%	21 406	23.4%	12 437	13.6%	4427	4.8%
	n	%	n	%	n	%	n	%
Age at delivery (years)								
<25	4298	8.1	1468	6.9	2398	19.3	828	18.7
25‐29	14 556	27.3	5251	24.5	4088	32.9	1253	28.3
30‐34	19 392	36.4	8018	37.5	3666	29.5	1291	29.2
35‐39	12 476	23.4	5541	25.9	1953	15.7	863	19.5
≥40	2632	4.9	1124	5.3	331	2.7	192	4.3
Mean (SD)	31.5	4.9	31.9	4.8	29.4	5.3	30.0	5.6
Body mass index								
<18.5	8757	16.4	3048	14.3	2217	17.8	820	18.5
18.5‐<25	39 590	74.2	15 858	74.1	8629	69.4	2913	65.9
≥25	4991	9.4	2490	11.6	1584	12.7	690	15.6
Diabetes mellitus history								
Yes	413	0.8	235	1.1	108	0.9	50	1.1
Marital status								
Married	51 570	97.0	20 678	96.9	11 085	89.8	3738	85.8
Single	1619	3.0	661	3.1	1259	10.2	619	14.2
Education history (years)								
<13	13 707	26.2	8464	40.4	7037	58.1	3109	73.2
13‐14	23 338	44.6	9208	44.0	4213	34.8	1007	23.7
≥15	15 291	29.2	3257	15.6	866	7.2	131	3.1
Annual household income (yen)								
<4 million	16 903	34.4	8253	41.9	5994	54.4	2385	61.7
4‐<6 million	16 785	34.2	6698	34.0	3208	29.1	979	25.3
≥6 million	15 463	31.5	4727	24.0	1813	16.5	500	12.9
*In vitro* fertilization or artificial insemination								
Yes	4100	7.7	1584	7.4	345	2.8	63	1.4
Alcohol intake								
Never drank	21 915	41.2	5319	24.9	3163	25.6	1063	24.2
Quit before pregnancy	25 930	48.8	13 352	62.6	8423	68.1	2850	64.8
Full drinking	5289	10.0	2656	12.5	789	6.4	485	11.0
Frequency of tobacco smoke exposure								
Never	31 644	59.5	10 613	49.7	2371	19.2	395	9.0
1 day a week	7151	13.5	2899	13.6	1011	8.2	179	4.1
2‐3 days a week	5615	10.6	2683	12.6	1593	12.9	461	10.5
4‐6 days a week	3732	7.0	1816	8.5	1703	13.8	519	11.8
Everyday	5012	9.4	3324	15.6	5699	46.1	2842	64.7
Folic acid intake								
Yes	27 245	51.2	10 858	50.9	5383	43.4	1463	33.2
Antihypertensive intake								
Yes	265	0.5	123	0.6	69	0.6	30	0.7
Anti‐convulsant intake								
Yes	179	0.3	89	0.4	71	0.6	32	0.7
Retinoic acid intake								
Yes	142	0.3	60	0.3	30	0.2	6	0.1

Table 3 shows the ORs from the logistic regression analysis of each smoking category (using “never smoked” as the reference) for the eight main CA groups as well as the “any CAs” category. Compared with never‐smokers, the OR was significantly elevated in the full‐smoking group for trisomy (adjusted OR, 2.14; 95% CI, 1.15‐3.97) and “any CAs” (adjusted OR, 1.35; 95% CI, 1.09‐1.67). No significant elevation was found in the QBP, QAP, or full‐smoking groups for CAs related to the nervous system; eyes, ears, face, and neck; cardiovascular system; oral clefts; digestive system; genital organs; or musculoskeletal system.

**TABLE 3:** Association between maternal smoking history and congenital anomaly group

ICD‐10 code	n (%)	Crude	Adjusted^a^
		OR (95% CI)	OR^a^ (95% CI)
Nervous system (Q00‐07)			
Never smoked	100 (0.19)	Reference	Reference
Quit before pregnancy	43 (0.20)	1.07 (0.75, 1.53)	1.06 (0.70, 1.61)
Quit after pregnancy	18 (0.14)	0.77 (0.47, 1.28)	0.76 (0.40, 1.43)
Full smoking	12 (0.27)	1.45 (0.80, 2.64)	1.42 (0.66, 3.07)
Eye, ear, face, and neck (Q10‐18)			
Never smoked	38 (0.07)	Reference	Reference
Quit before pregnancy	13 (0.06)	0.85 (0.45, 1.60)	0.96 (0.49, 1.89)
Quit after pregnancy	8 (0.06)	0.90 (0.42, 1.94)	1.06 (0.45, 2.51)
Full smoking^b^	4 (0.09)	–	–
Cardiovascular system (Q20‐28)			
Never smoked	616 (1.15)	Reference	Reference
Quit before pregnancy	239 (1.12)	0.97 (0.83, 1.12)	1.00 (0.85, 1.17)
Quit after pregnancy	139 (1.12)	0.97 (0.80, 1.16)	0.98 (0.79, 1.22)
Full smoking	64 (1.45)	1.26 (0.97, 1.63)	1.34 (0.99, 1.82)
Oral clefts (Q35‐37)			
Never smoked	128 (0.24)	Reference	Reference
Quit before pregnancy	58 (0.27)	1.13 (0.83, 1.54)	1.16 (0.82, 1.64)
Quit after pregnancy	33 (0.27)	1.11 (0.76, 1.62)	1.17 (0.74, 1.85)
Full smoking	10 (0.23)	0.94 (0.49, 1.79)	1.17 (0.58, 2.38)
Digestive system (Q38‐45)			
Never smoked	55 (0.10)	Reference	Reference
Quit before pregnancy	31 (0.14)	1.41 (0.91, 2.18)	1.58 (0.98, 2.55)
Quit after pregnancy^b^	2 (0.02)	–	–
Full smoking^b^	3 (0.07)	–	–
Genital organs (Q50‐56)^c^			
Never smoked	190 (0.70)	Reference	Reference
Quit before pregnancy	79 (0.72)	1.04 (0.80, 1.35)	0.99 (0.74, 1.32)
Quit after pregnancy	38 (0.60)	0.86 (0.61, 1.22)	0.80 (0.53, 1.22)
Full smoking	21 (0.92)	1.33 (0.84, 2.08)	1.22 (0.71, 2.11)
Musculoskeletal system (Q65‐79)			
Never smoked	185 (0.35)	Reference	Reference
Quit before pregnancy	86 (0.40)	1.16 (0.90, 1.50)	1.18 (0.89, 1.57)
Quit after pregnancy	38 (0.31)	0.88 (0.62, 1.25)	0.93 (0.62, 1.41)
Full smoking	23 (0.52)	1.50 (0.97, 2.32)	1.40 (0.81, 2.43)
Trisomy (Q90‐91)			
Never smoked	110 (0.21)	Reference	Reference
Quit before pregnancy	40 (0.19)	0.91 (0.63, 1.30)	0.75 (0.49, 1.15)
Quit after pregnancy	14 (0.11)	0.55 (0.31, 0.95)	0.55 (0.28, 1.10)
Full smoking	18 (0.41)	**1.98 (1.20, 3.26)**	**2.14 (1.15, 3.97)**
Any congenital anomalies			
Never smoked	1263 (2.37)	Reference	Reference
Quit before pregnancy	526 (2.46)	1.04 (0.94, 1.15)	1.06 (0.95, 1.19)
Quit after pregnancy	273 (2.20)	0.93 (0.81, 1.06)	0.92 (0.79, 1.08)
Full smoking	137 (3.09)	**1.32 (1.10, 1.58)**	**1.35 (1.09, 1.67)**

Abbreviations: CI, confidence interval; OR, odds ratio.

^a^ Adjusted for maternal age at delivery, body mass index, history of diabetes mellitus, marital status, education history, annual household income, *in vitro* fertilization or artificial insemination, alcohol intake, frequency of tobacco smoke exposure, folic acid intake, antihypertensive intake, anti‐convulsant intake, and retinoic acid intake.

^b^ We did not calculate the odds ratio because a number of cases were too small (<5 cases).

^c^ Only boys were analyzed (n = 46 893).

Table 4 shows the ORs from the logistic regression analysis of each smoking category for the 10 anomalies. Compared with never‐smokers, the OR in the full‐smoking group was significantly elevated for only Down syndrome (adjusted OR, 2.01; 95% CI, 1.01‐4.25).

**TABLE 4:** Association between maternal smoking history and frequent congenital anomalies in this study

Anomalies	n (%)	Crude	Adjusted^a^
		OR (95% CI)	OR^a^ (95% CI)
Hydrocephaly			
Never smoked	44(0.08)	Reference	Reference
Quit before pregnancy	19(0.09)	1.08 (0.63, 1.84)	1.22 (0.67, 2.22)
Quit after pregnancy	10(0.08)	0.98 (0.49, 1.94)	1.13 (0.49, 2.62)
Full smoking	8(0.18)	2.19 (1.03, 4.66)	2.55 (0.95, 6.86)
Cleft palate			
Never smoked	34 (0.06)	Reference	Reference
Quit before pregnancy	11 (0.05)	0.81 (0.41, 1.59)	0.92 (0.45, 1.88)
Quit after pregnancy	5 (0.04)	0.63 (0.25, 1.61)	0.88 (0.31, 2.50)
Full smoking^b^	1 (0.02)	–	–
Cleft lip			
Never smoked	38 (0.07)	Reference	Reference
Quit before pregnancy	20 (0.09)	1.31 (0.76, 2.26)	1.25 (0.66, 2.37)
Quit after pregnancy	10 (0.08)	1.13 (0.56, 2.27)	1.18 (0.50, 2.80)
Full smoking	6 (0.14)	1.90 (0.81, 4.51)	2.40 (0.87, 6.59)
Cleft lip and palate			
Never smoked	63 (0.12)	Reference	Reference
Quit before pregnancy	28 (0.13)	1.11 (0.71, 1.73)	1.14 (0.70, 1.86)
Quit after pregnancy	21 (0.17)	1.43 (0.87, 2.35)	1.32 (0.72, 2.42)
Full smoking^b^	4 (0.09)	–	–
Cryptorchidism^c^			
Never smoked	161 (0.59)	Reference	Reference
Quit before pregnancy	68 (0.62)	1.05 (0.79, 1.40)	0.98 (0.72, 1.33)
Quit after pregnancy	32 (0.51)	0.86 (0.59, 1.26)	0.80 (0.51, 1.25)
Full smoking	18 (0.79)	1.34 (0.82, 2.19)	1.14 (0.63, 2.08)
Hypospadias^c^			
Never smoked	33 (0.12)	Reference	Reference
Quit before pregnancy	15 (0.14)	1.13 (0.62, 2.09)	1.27 (0.65, 2.47)
Quit after pregnancy	7 (0.11)	0.92 (0.41, 2.07)	0.91 (0.32, 2.57)
Full smoking^b^	3 (0.13)	–	–
Polydactyly of fingers			
Never smoked	57 (0.11)	Reference	Reference
Quit before pregnancy	27 (0.13)	1.18 (0.75, 1.87)	1.07 (0.64, 1.79)
Quit after pregnancy	12 (0.10)	0.90 (0.49, 1.68)	0.88 (0.43, 1.79)
Full smoking	9 (0.20)	1.91 (0.94, 3.85)	1.58 (0.66, 3.80)
Polydactyly of toes			
Never smoked	46 (0.09)	Reference	Reference
Quit before pregnancy	21 (0.10)	1.14 (0.68, 1.91)	1.25 (0.73, 2.14)
Quit after pregnancy	12 (0.10)	1.12 (0.59, 2.11)	1.17 (0.55, 2.49)
Full smoking^b^	4 (0.09)	–	–
Syndactyly of toes			
Never smoked	53 (0.10)	Reference	Reference
Quit before pregnancy	22 (0.10)	1.04 (0.63, 1.71)	1.14 (0.66, 1.97)
Quit after pregnancy	9 (0.07)	0.73 (0.36, 1.48)	0.76 (0.32, 1.78)
Full smoking	6 (0.14)	1.37 (0.59, 3.18)	1.30 (0.43, 3.96)
Down syndrome			
Never smoked	85 (0.16)	Reference	Reference
Quit before pregnancy	30 (0.14)	0.88 (0.58, 1.33)	0.75 (0.46, 1.23)
Quit after pregnancy	11 (0.09)	0.56 (0.30, 1.04)	0.58 (0.27, 1.26)
Full smoking	13 (0.29)	**1.85 (1.03, 3.31)**	**2.08 (1.01, 4.25)**

Abbreviations: CI, confidence interval; OR, odds ratio.

^a^ Adjusted for maternal age at delivery, body mass index, history of diabetes mellitus, marital status, education history, annual household income, *in vitro* fertilization or artificial insemination, alcohol intake, frequency of tobacco smoke exposure, folic acid intake, antihypertensive intake, anti‐convulsant intake, and retinoic acid intake.

^b^ We did not calculate the odds ratio because the number of cases was too small (<5 cases).

^c^ Only boys were analyzed (n = 46 893).

## 4 DISCUSSION

In this study, we showed that the risk of any CAs and trisomy was significantly increased in the full‐smoking during pregnancy group compared with the never smoked group. We recruited pregnant women who visited general obstetric clinics, which covered nearly 50% of all births in the 15 different regions throughout Japan.^18^ Data on CAs were collected by transcribing information from medical records at two time points: at birth and at 1 month postpartum. We collected records on abortion and stillborn infants as well as live infants.^20^ In addition, we found that several indicators are similar to those of the 2013 National Vital Statistics Survey,^21^ and we therefore believe that our results are representative of the general population in Japan.^19^ To our knowledge, the present study is the first to report on the association of maternal smoking history and CA risk in a Japan‐wide birth cohort.

On the other hand, except for trisomy, there were no significant associations between the full‐smoking group and CAs related to the nervous system; eyes, ears, face, and neck; cardiovascular system; oral clefts; genital organs; and the musculoskeletal system. For CAs of the cardiovascular system, the OR was higher in the full‐smoking group, but not significantly (1.34; 95% CI, 0.99‐1.82). A systematic review that analyzed 43 studies reported that smoking during pregnancy increases the risk of congenital heart disease (CHD).^9^ The association between the effect of tobacco smoke exposure during pregnancy and CHD has not been fully elucidated, but there are reports of genetic polymorphisms that increase the risk of CHD in children whose mothers were exposed to tobacco smoke.^22^, ^23^ Although our results on CHD were not statistically significant, we believe that maternal smoking increases the risk of CHD. Half of the CAs assessed in this study were CHD, which might have had a major impact on the overall results.

Given that many subcategories were included in the “any CAs” category, these abnormalities might have occurred at various stages of embryological development. In general, most CAs occur during organ formation (between the 4th and 7th week of pregnancy).^24^ Although some mothers notice their pregnancy during this early stage, many do not; hence, it is uncertain why the findings could be so different for the QAP group (i.e., those who quit smoking only after realizing they were pregnant). However, even after becoming pregnant, quitting smoking might be preventive against the occurrence of CAs in fetuses. In two birth cohort studies, smoking during pregnancy was found to increase the incidence of any CAs.^15^, ^16^ To trace associations between these previous studies and the present study, we examined the risk of CAs in the combined QAP and full‐smoking groups as a single “smoking during conception” group, but found no significant associations for any categories of anomalies (Table S1) or frequent congenital anomalies (Table S2). This finding might indicate the possibility of low tobacco exposure in the QAP group; indeed, none of the CAs observed in the QAP group showed a significant association. The frequency of maternal smoking during pregnancy in the Danish register‐based cohort study was 18.6%,^15^ which is nearly the same as the rate of 18.4% in the combined QAP and full‐smoking groups in the JECS; however, in the Danish study, only 11.5% of mothers who smoked quit during pregnancy compared with 73.7% in the JECS. This also suggests that the QAP group in the JECS might have been less dependent on tobacco; hence, the exposure might have been weaker for the same reason. Therefore, it is not possible to determine from our study whether quitting smoking after pregnancy is preventive. We hope that this will be verified in future trials of smoking cessation interventions for pregnant women.

For trisomy, our results showed increased risk in the full‐smoking group, but some previous studies have shown no significant associations between maternal smoking and trisomy^14^, ^15^ or have shown low ORs for trisomy in children of smokers.^25^, ^26^ Therefore, the results of the present study need to be interpreted carefully. Trisomy zygotes are primarily a result of maternal meiotic I errors, and the risk of oocyte aneuploidy increases with age.^27^, ^28^ The mechanism that gives rise to oocyte aneuploidy with aging has not yet been elucidated, but some of the molecular pathways are becoming clearer and age‐related degradation of cohesin has been suggested as a possible mechanism.^29^ Along with aging, long‐term exposure to tobacco smoke might be related to a molecular mechanism such as degradation of cohesin in the full‐smoking group. In addition, compared with the other groups, the full‐smoking group might be more susceptible to oocyte degradation because they had more tobacco smoke exposure as a result of their partner's or the other family member's smoking habit (Table S3). Thus, further research is needed to elucidate whether long‐term exposure to tobacco smoke might be related to oocyte degradation. However, we cannot deny the possibility of bias induced by artificial abortion as a result of prenatal diagnosis of trisomy. Compared with the full‐smoking group, it seems that it is easier to approach the non‐smoking group in order to investigate trisomy‐specific abortions because the women in this group have higher income and higher frequency of using assisted reproductive technologies (in vitro fertilization and/or artificial insemination). Although our analysis included cases of abortion, some women might have withdrawn from the study after an abortion and their records would be unavailable for analysis.

In addition to the aforementioned limitation, there are some further limitations in this study. First, the data we collected related to smoking histories and quantity were self‐reported and therefore could not be analyzed as objective measures. In previous studies based on cotinine concentrations detected in blood, some mothers who self‐reported that they had “never smoked” were evidently “full smoking.”^30^, ^31^ In the present study, the never smoked and QBP groups might have included participants who gave false or mistaken answers. Second, this study used the 31 ailments and abnormalities defined by the JECS, and thus the analysis was conducted based on these categories. The strength of this study is that it is based on a nationwide survey conducted in Japan with a very high response rate^19^ from the start of the study (during pregnancy) to the time of delivery. Many responses related to smoking behaviors were obtained. Multiple covariates (confounding factors) regarded as optimal for analysis were available in the study, thereby enabling many different effects to be considered and discarded.

## 5 CONCLUSIONS

This study showed that children born to women who continue to smoke during pregnancy have increased risk of trisomy and CAs. We eagerly await further studies that will clarify the etiology and underlying mechanisms that induce CAs due to maternal smoking.

## CONFLICT OF INTEREST

The authors declare no conflict of interest.

## Supporting information

Supporting Information

**TABLE S1**: Association between maternal smoking during conception and congenital anomaly group

**TABLE S2**: Association between maternal smoking during conception and frequent congenital anomalies in this study

**TABLE S3**: Other smoking statuses according to maternal smoking history

## APPENDIX A.

Members of the JECS Group as of 2021: Michihiro Kamijima (principal investigator, Nagoya City University, Nagoya, Japan), Shin Yamazaki (National Institute for Environmental Studies, Tsukuba, Japan), Yukihiro Ohya (National Center for Child Health and Development, Tokyo, Japan), Reiko Kishi (Hokkaido University, Sapporo, Japan), Nobuo Yaegashi (Tohoku University, Sendai, Japan), Koichi Hashimoto (Fukushima Medical University, Fukushima, Japan), Chisato Mori (Chiba University, Chiba, Japan), Shuichi Ito (Yokohama City University, Yokohama, Japan), Zentaro Yamagata (University of Yamanashi, Chuo, Japan), Hidekuni Inadera (University of Toyama, Toyama, Japan), Takeo Nakayama (Kyoto University, Kyoto, Japan), Hiroyasu Iso (Osaka University, Suita, Japan), Masayuki Shima (Hyogo College of Medicine, Nishinomiya, Japan), Youichi Kurozawa (Tottori University, Yonago, Japan), Narufumi Suganuma (Kochi University, Nankoku, Japan), Koichi Kusuhara (University of Occupational and Environmental Health, Kitakyushu, Japan), and Takahiko Katoh (Kumamoto University, Kumamoto, Japan).
