# Supplementary material for: Association between maternal smoking history and congenital anomalies in children: Results from the Japan Environment and Children's Study
Source: Congenit Anom (Kyoto). 2021 Jun 8;61(5):159–68. doi: 10.1111/cga.12430 (PMC8453515; doi:10.1111/cga.12430)
Supplement: Supplementary file 1 — Supporting Information [file CGA-61-159-s002.docx]

***Congenital Anomalies* Form for Disclosure of Potential Conflicts of Interest**

**Section 1. Identifying Information**

1. Given Name (First Name) 2. Surname (Last Name) 3. Effective Date

Akiko 　　　 Tsuchida 19 April 2021

4. Are you the corresponding author? □Yes　　☑ No

(Name of the corresponding author)　　　　Hidekuni Inadera

5. Manuscript Title

Association between maternal smoking history and congenital anomalies in children: results from the Japan Environment and Children's Study

6. Manuscript Identifying Number (if you know it)

**Section 2. The Work under Consideration for Publication**

Did you or your institution at any time receive payment or services from a third party for any aspect of the submitted work (including but not limited to grants, data monitoring board, study design, manuscript preparation, statistical analysis, etc…)?

Complete each row by checking “No” or providing the requested information.

**The Work Under Consideration for Publication**

| Type | No | Money Paid to You | Money to Your Institution* | Name of Entity | Comments** |
| --- | --- | --- | --- | --- | --- |
| 1. Grant | ☑ | □ | □ |  |  |
|  |  |  |  |  |  |
| 2. Consulting fee or honorarium | ☑ | □ | □ |  |  |
|  |  |  |  |  |  |
| 3. Support for travel to meetings for the study or other purposes | ☑ | □ | □ |  |  |
|  |  |  |  |  |  |
| 4. Fees for participation in review activities such as data monitoring boards, statistical analysis, end point committees, and the like | ☑ | □ | □ |  |  |
|  |  |  |  |  |  |
| 5. Payment for writing or reviewing　the manuscript | ☑ | □ | □ |  |  |
|  |  |  |  |  |  |
| 6. Provision of writing assistance, medicines, equipment, or administrative support | ☑ | □ | □ |  |  |
|  |  |  |  |  |  |
| 7. Other | ☑ | □ | □ |  |  |
|  |  |  |  |  |  |

* This means money that your institution received for your efforts on this study.

** Use this section to provide any needed explanation.

**Section 3. Other relationships**

Are there other relationships or activities that readers could perceive to have influenced, or that give the appearance of potentially influencing, what you wrote in the submitted work?

☑ No other relationships/conditions/circumstances that present a potential conflict of interest

□ Yes, the following relationships/conditions/circumstances are present (explain below):

At the time of manuscript acceptance, journals will ask authors to confirm and, if necessary, update their disclosure statements.

On occasion, journals may ask authors to disclose further information about reported relationships.

***Congenital Anomalies* Form for Disclosure of Potential Conflicts of Interest**

**Section 1. Identifying Information**

1. Given Name (First Name) 2. Surname (Last Name) 3. Effective Date

Kei 　　　 Hamazaki 19 April 2021

4. Are you the corresponding author? □Yes　　☑ No

(Name of the corresponding author)　　　　Hidekuni Inadera

5. Manuscript Title

Association between maternal smoking history and congenital anomalies in children: results from the Japan Environment and Children's Study

6. Manuscript Identifying Number (if you know it)

**Section 2. The Work under Consideration for Publication**

Did you or your institution at any time receive payment or services from a third party for any aspect of the submitted work (including but not limited to grants, data monitoring board, study design, manuscript preparation, statistical analysis, etc…)?

Complete each row by checking “No” or providing the requested information.

**The Work Under Consideration for Publication**

| Type | No | Money Paid to You | Money to Your Institution* | Name of Entity | Comments** |
| --- | --- | --- | --- | --- | --- |
| 1. Grant | ☑ | □ | □ |  |  |
|  |  |  |  |  |  |
| 2. Consulting fee or honorarium | ☑ | □ | □ |  |  |
|  |  |  |  |  |  |
| 3. Support for travel to meetings for the study or other purposes | ☑ | □ | □ |  |  |
|  |  |  |  |  |  |
| 4. Fees for participation in review activities such as data monitoring boards, statistical analysis, end point committees, and the like | ☑ | □ | □ |  |  |
|  |  |  |  |  |  |
| 5. Payment for writing or reviewing　the manuscript | ☑ | □ | □ |  |  |
|  |  |  |  |  |  |
| 6. Provision of writing assistance, medicines, equipment, or administrative support | ☑ | □ | □ |  |  |
|  |  |  |  |  |  |
| 7. Other | ☑ | □ | □ |  |  |
|  |  |  |  |  |  |

* This means money that your institution received for your efforts on this study.

** Use this section to provide any needed explanation.

**Section 3. Other relationships**

Are there other relationships or activities that readers could perceive to have influenced, or that give the appearance of potentially influencing, what you wrote in the submitted work?

☑ No other relationships/conditions/circumstances that present a potential conflict of interest

□ Yes, the following relationships/conditions/circumstances are present (explain below):

At the time of manuscript acceptance, journals will ask authors to confirm and, if necessary, update their disclosure statements.

On occasion, journals may ask authors to disclose further information about reported relationships.

***Congenital Anomalies* Form for Disclosure of Potential Conflicts of Interest**

**Section 1. Identifying Information**

1. Given Name (First Name) 2. Surname (Last Name) 3. Effective Date

Mika 　　　 Kigawa 19 April 2021

4. Are you the corresponding author? □Yes　　☑ No

(Name of the corresponding author)　　　　Hidekuni Inadera

5. Manuscript Title

Association between maternal smoking history and congenital anomalies in children: results from the Japan Environment and Children's Study

6. Manuscript Identifying Number (if you know it)

**Section 2. The Work under Consideration for Publication**

Did you or your institution at any time receive payment or services from a third party for any aspect of the submitted work (including but not limited to grants, data monitoring board, study design, manuscript preparation, statistical analysis, etc…)?

Complete each row by checking “No” or providing the requested information.

**The Work Under Consideration for Publication**

| Type | No | Money Paid to You | Money to Your Institution* | Name of Entity | Comments** |
| --- | --- | --- | --- | --- | --- |
| 1. Grant | ☑ | □ | □ |  |  |
|  |  |  |  |  |  |
| 2. Consulting fee or honorarium | ☑ | □ | □ |  |  |
|  |  |  |  |  |  |
| 3. Support for travel to meetings for the study or other purposes | ☑ | □ | □ |  |  |
|  |  |  |  |  |  |
| 4. Fees for participation in review activities such as data monitoring boards, statistical analysis, end point committees, and the like | ☑ | □ | □ |  |  |
|  |  |  |  |  |  |
| 5. Payment for writing or reviewing　the manuscript | ☑ | □ | □ |  |  |
|  |  |  |  |  |  |
| 6. Provision of writing assistance, medicines, equipment, or administrative support | ☑ | □ | □ |  |  |
|  |  |  |  |  |  |
| 7. Other | ☑ | □ | □ |  |  |
|  |  |  |  |  |  |

* This means money that your institution received for your efforts on this study.

** Use this section to provide any needed explanation.

**Section 3. Other relationships**

Are there other relationships or activities that readers could perceive to have influenced, or that give the appearance of potentially influencing, what you wrote in the submitted work?

☑ No other relationships/conditions/circumstances that present a potential conflict of interest

□ Yes, the following relationships/conditions/circumstances are present (explain below):

At the time of manuscript acceptance, journals will ask authors to confirm and, if necessary, update their disclosure statements.

On occasion, journals may ask authors to disclose further information about reported relationships.

***Congenital Anomalies* Form for Disclosure of Potential Conflicts of Interest**

**Section 1. Identifying Information**

1. Given Name (First Name) 2. Surname (Last Name) 3. Effective Date

Tomomi 　　　 Tanaka 19 April 2021

4. Are you the corresponding author? □Yes　　☑ No

(Name of the corresponding author)　　　　Hidekuni Inadera

5. Manuscript Title

Association between maternal smoking history and congenital anomalies in children: results from the Japan Environment and Children's Study

6. Manuscript Identifying Number (if you know it)

**Section 2. The Work under Consideration for Publication**

Did you or your institution at any time receive payment or services from a third party for any aspect of the submitted work (including but not limited to grants, data monitoring board, study design, manuscript preparation, statistical analysis, etc…)?

Complete each row by checking “No” or providing the requested information.

**The Work Under Consideration for Publication**

| Type | No | Money Paid to You | Money to Your Institution* | Name of Entity | Comments** |
| --- | --- | --- | --- | --- | --- |
| 1. Grant | ☑ | □ | □ |  |  |
|  |  |  |  |  |  |
| 2. Consulting fee or honorarium | ☑ | □ | □ |  |  |
|  |  |  |  |  |  |
| 3. Support for travel to meetings for the study or other purposes | ☑ | □ | □ |  |  |
|  |  |  |  |  |  |
| 4. Fees for participation in review activities such as data monitoring boards, statistical analysis, end point committees, and the like | ☑ | □ | □ |  |  |
|  |  |  |  |  |  |
| 5. Payment for writing or reviewing　the manuscript | ☑ | □ | □ |  |  |
|  |  |  |  |  |  |
| 6. Provision of writing assistance, medicines, equipment, or administrative support | ☑ | □ | □ |  |  |
|  |  |  |  |  |  |
| 7. Other | ☑ | □ | □ |  |  |
|  |  |  |  |  |  |

* This means money that your institution received for your efforts on this study.

** Use this section to provide any needed explanation.

**Section 3. Other relationships**

Are there other relationships or activities that readers could perceive to have influenced, or that give the appearance of potentially influencing, what you wrote in the submitted work?

☑ No other relationships/conditions/circumstances that present a potential conflict of interest

□ Yes, the following relationships/conditions/circumstances are present (explain below):

At the time of manuscript acceptance, journals will ask authors to confirm and, if necessary, update their disclosure statements.

On occasion, journals may ask authors to disclose further information about reported relationships.

***Congenital Anomalies* Form for Disclosure of Potential Conflicts of Interest**

**Section 1. Identifying Information**

1. Given Name (First Name) 2. Surname (Last Name) 3. Effective Date

Mika 　　　 　　 Ito 19 April 2021

4. Are you the corresponding author? □Yes　　☑ No

(Name of the corresponding author)　　　　Hidekuni Inadera

5. Manuscript Title

Association between maternal smoking history and congenital anomalies in children: results from the Japan Environment and Children's Study

6. Manuscript Identifying Number (if you know it)

**Section 2. The Work under Consideration for Publication**

Did you or your institution at any time receive payment or services from a third party for any aspect of the submitted work (including but not limited to grants, data monitoring board, study design, manuscript preparation, statistical analysis, etc…)?

Complete each row by checking “No” or providing the requested information.

**The Work Under Consideration for Publication**

| Type | No | Money Paid to You | Money to Your Institution* | Name of Entity | Comments** |
| --- | --- | --- | --- | --- | --- |
| 1. Grant | ☑ | □ | □ |  |  |
|  |  |  |  |  |  |
| 2. Consulting fee or honorarium | ☑ | □ | □ |  |  |
|  |  |  |  |  |  |
| 3. Support for travel to meetings for the study or other purposes | ☑ | □ | □ |  |  |
|  |  |  |  |  |  |
| 4. Fees for participation in review activities such as data monitoring boards, statistical analysis, end point committees, and the like | ☑ | □ | □ |  |  |
|  |  |  |  |  |  |
| 5. Payment for writing or reviewing　the manuscript | ☑ | □ | □ |  |  |
|  |  |  |  |  |  |
| 6. Provision of writing assistance, medicines, equipment, or administrative support | ☑ | □ | □ |  |  |
|  |  |  |  |  |  |
| 7. Other | ☑ | □ | □ |  |  |
|  |  |  |  |  |  |

* This means money that your institution received for your efforts on this study.

** Use this section to provide any needed explanation.

**Section 3. Other relationships**

Are there other relationships or activities that readers could perceive to have influenced, or that give the appearance of potentially influencing, what you wrote in the submitted work?

☑ No other relationships/conditions/circumstances that present a potential conflict of interest

□ Yes, the following relationships/conditions/circumstances are present (explain below):

At the time of manuscript acceptance, journals will ask authors to confirm and, if necessary, update their disclosure statements.

On occasion, journals may ask authors to disclose further information about reported relationships.

***Congenital Anomalies* Form for Disclosure of Potential Conflicts of Interest**

**Section 1. Identifying Information**

1. Given Name (First Name) 2. Surname (Last Name) 3. Effective Date

Hidekuni 　　　 　　 Inadera 19 April 2021

4. Are you the corresponding author? ☑Yes　　□ No

(Name of the corresponding author)　　　　Hidekuni Inadera

5. Manuscript Title

Association between maternal smoking history and congenital anomalies in children: results from the Japan Environment and Children's Study

6. Manuscript Identifying Number (if you know it)

**Section 2. The Work under Consideration for Publication**

Did you or your institution at any time receive payment or services from a third party for any aspect of the submitted work (including but not limited to grants, data monitoring board, study design, manuscript preparation, statistical analysis, etc…)?

Complete each row by checking “No” or providing the requested information.

**The Work Under Consideration for Publication**

| Type | No | Money Paid to You | Money to Your Institution* | Name of Entity | Comments** |
| --- | --- | --- | --- | --- | --- |
| 1. Grant | ☑ | □ | □ |  |  |
|  |  |  |  |  |  |
| 2. Consulting fee or honorarium | ☑ | □ | □ |  |  |
|  |  |  |  |  |  |
| 3. Support for travel to meetings for the study or other purposes | ☑ | □ | □ |  |  |
|  |  |  |  |  |  |
| 4. Fees for participation in review activities such as data monitoring boards, statistical analysis, end point committees, and the like | ☑ | □ | □ |  |  |
|  |  |  |  |  |  |
| 5. Payment for writing or reviewing　the manuscript | ☑ | □ | □ |  |  |
|  |  |  |  |  |  |
| 6. Provision of writing assistance, medicines, equipment, or administrative support | ☑ | □ | □ |  |  |
|  |  |  |  |  |  |
| 7. Other | ☑ | □ | □ |  |  |
|  |  |  |  |  |  |

* This means money that your institution received for your efforts on this study.

** Use this section to provide any needed explanation.

**Section 3. Other relationships**

Are there other relationships or activities that readers could perceive to have influenced, or that give the appearance of potentially influencing, what you wrote in the submitted work?

☑ No other relationships/conditions/circumstances that present a potential conflict of interest

□ Yes, the following relationships/conditions/circumstances are present (explain below):

At the time of manuscript acceptance, journals will ask authors to confirm and, if necessary, update their disclosure statements.

On occasion, journals may ask authors to disclose further information about reported relationships.
